# The Impact of the COVID-19 Pandemic on Influenza Transmission in Poland

**DOI:** 10.3390/microorganisms11040970

**Published:** 2023-04-08

**Authors:** Katarzyna Łuniewska, Karol Szymański, Katarzyna Kondratiuk, Ewelina Hallmann, Lidia Bernadeta Brydak

**Affiliations:** National Influenza Centre, Department of Influenza Research, National Institute of Public Health NIH—National Research Institute, 00-791 Warsaw, Poland

**Keywords:** influenza, COVID-19, virology, epidemic season, pandemic, surveillance

## Abstract

Background: The aim of this study was to determine whether the occurrence of the SARS-CoV-2 pandemic affected the incidence of influenza in Poland and the efficiency of the SENTINEL influenza surveillance system. Methods: The analysis was based on virologic data from the 2018/2019–2021/2022 epidemic seasons. The data in question were obtained from the SENTINEL influenza surveillance system, which is utilized in Poland. Results: In the 2020/2021 epidemic season, only one positive case was confirmed. In the epidemic season of 2021/2022, the number of positive cases increased. There was a delay in the peak of the season, since the start of pandemic, which was observed in the 14th week of 2022. Previously, it was recorded in the 5–10th week, depending on the season. Before the pandemic, the number of positive samples in relation to the tested ones oscillated between 41–49.4%. After the pandemic, it was 0.3% and below 20%, respectively, for season 2020/2021 and season 2021/2022. Conclusions: The COVID-19 pandemic caused a decline in many other infectious diseases, including influenza, as a result of the numerous lockdowns and from people shifting to remote work. Other safety measures, such as obligatory protective masks and the use of disinfectants, had a significant impact on reducing the number of cases.

## 1. Introduction

In 2019, China encountered an increase in the incidence of pneumonia of unknown etiology. The origin of this phenomenon was quickly found. It was identified as a new coronavirus, later named Severe Acute Respiratory Syndrome Coronavirus 2 (SARS-CoV-2) [1]. The disease entity was defined as COVID-19. On 20 March 2020, the World Health Organization classified the spreading the virus as a pandemic [2]. Many restrictions were introduced to minimize the contagion of SARS-CoV-2. Many governments imposed travel restrictions. Companies introduced the possibility of remote work. Schools were closed, with classes held only in online mode. Citizens were also obligated to wear protective masks. The use of disinfectants that prevent the spread of SARS-CoV-2 also become popular. They were effective and inexpensive, which allowed them to be used for personal protection as well as in stores, healthcare facilities, and offices [3]. Such activities did not necessarily prevent the spread of other diseases, including influenza.

Influenza is a contagious viral disease. Infection occurs by droplet and aerosol infection through contact with an infected person. Epidemics are observed every season, during which 3–5 million people fall ill [4]. They are caused by influenza A viruses (A/H1N1/pdm09, A/H3N2/) and influenza B viruses (B/Victoria lineage, B/Yamagata lineage) belonging to the family Orthomyxoviridae [5]. Influenza symptoms are very non-specific. The most common of these are a fever above 38 °C, muscle pain, cough, and sore throat. The prevention of influenza is mainly based on the use of seasonal influenza vaccination. It prevents both illnesses and severe symptoms and complications [6].

Both COVID-19 and influenza have a significant impact not only on public health, but also on the environment, the global economy, and the education system. The COVID-19 pandemic was cited as causing the most profound public health crisis in more than century [7]. There were justified concerns that COVID-19, influenza, and other respiratory viruses would be circulating at the same time, putting a heavy strain on the healthcare system. However, instead of having several respiratory viruses spread simultaneously, it was observed that the infectivity of the influenza virus was significantly reduced [8]. This tendency could be observed in Poland thanks to the functioning of the SENTINEL influenza surveillance system.

Rapid confirmation of the influenza infection is of great importance for public health due to the availability of next-generation anti-influenza drugs, which should be administered as soon as possible. Such a procedure is applied to protect the patient against complications from influenza, but also in order to prevent the transmission of the virus to the environment [6].

Influenza prevention has been a key public health challenge for years. When analyzing seasonal influenza epidemics, a combination of direct and indirect effects on both public health and economic effects should be considered [9]. Seasonal vaccination is the cheapest and most effective form of prophylaxis [6]. The higher the percentage of immunization of the population, the lower the number of visits to primary care physicians, hospitalizations, and deaths, which also reduces the costs borne by the state [10]. Many studies show that increasing the vaccination rate among children can bring both health and economic benefits. Reducing the number of influenza infections results in less frequent use of sick leave by parents, which reduces the losses incurred by employers [11]. In Poland, the level of vaccination of the population has been low for many years. There are currently three types of vaccines in Poland: split, subunit, and a live vaccine [12]. Due to the high variability of the influenza virus, the composition of the influenza vaccine changes every season. This composition is determined through research conducted at the National Influenza Centers, as well as at the WHO Collaborating Centers.

The aim of this study was to determine whether the occurrence of the SARS-CoV-2 pandemic had an impact on the number of influenza cases in Poland and the efficiency of the SENTINEL influenza surveillance system.

## 2. Materials and Methods

The analyses were based on data available as part of the SENTINEL system from the 2015/2016 epidemic season until the 2021/2022 season. Each analyzed sample was described by age, research method, and result, and was entered into the system by employees of the Voivodeship Sanitary Epidemiological Station (VSES). No specific testing method to confirm the presence of the influenza virus was imposed. The only condition was that it must be a molecular biology method. Laboratories perform the tests with the use of commercial kits in accordance with the manufacturer’s recommendations.

Poland has been a member of the Global Influenza Surveillance and Response System since 1957 [6]. Since 2003, measures have been taken to create an integrated influenza surveillance system. Since the 2004/2005 season, the tool used to monitor the virologic and epidemiological situation is the SENTINEL system. For each season, it covers the patients of over 500 general practitioners (GPs), which means that the monitoring includes approximately 1 million inhabitants of the country [13]. Surveillance is carried out throughout the influenza season. The increase in influenza activity in Poland is usually reported between January and April, which is in line with the trends in the northern hemisphere. The epidemiological and virological data are recorded on a weekly basis. Since the 2013/2014 epidemic season, reporting has been carried out with the use of an online platform. Epidemiological data include the numbers of patients with symptoms consistent with the adopted case definition of ILI (influenza-like illness) [14]. In contrast, virological data includes samples analyzed at laboratories using molecular biology methods. The data is collected in cooperation with 16 VSES and then aggregated, which makes it possible to define the overview of the situation in the entire country. The data is also represented by seven age groups (0–4, 5–9, 10–14, 15–25, 26–44, 45–64, 65+) to allow a more accurate assessment of the virological and epidemiological situation [15]. The Department of Influenza Research of the National influenza Centre (NIC) in Poland submits a weekly summary report to both the ECDC and the WHO. Thus, the global epidemiological situation is monitored on an ongoing basis, which ensures a quick response in the event of an influenza virus subtype with a possible pandemic potential.

VSES are required to send positive samples to the NIC for more detailed analyses two times a year. Most often they are based on the subtype of influenza A virus and influenza B lineage. We performed subtyping for all sent samples whose subtype was not specified. In the case of Influenza B, we specified the lineages. The VSES were not able to perform it in their laboratories. In the NIC, analyses are performed using primers and probes obtained from the International Reagent Resource in collaboration with the WHO. SuperScript Platinum III (Seegene) was used for the analysis. The reaction was carried out in a Rotor-Gene Q MDx 5 plex with the following reaction conditions: RNA was subjected to reverse transcription (50 °C, 30 min). The obtained DNA was subjected to the initial denaturation process (1 cycle at 95 °C for 2 min), followed by 45 cycles of amplification: denaturation at 95 °C for 15 s, annealing at 55 °C for 10 s, and elongation at 72 °C for 20 s. The final reaction volume was 20 uL:15 uL Master Mix and 5 uL isolated RNA. The positive control was viral RNA obtained from the strains contained in the vaccine, and the negative control was RNAse-free water.

## 3. Results

### 3.1. Peak of Incidence

**Figure 1:**
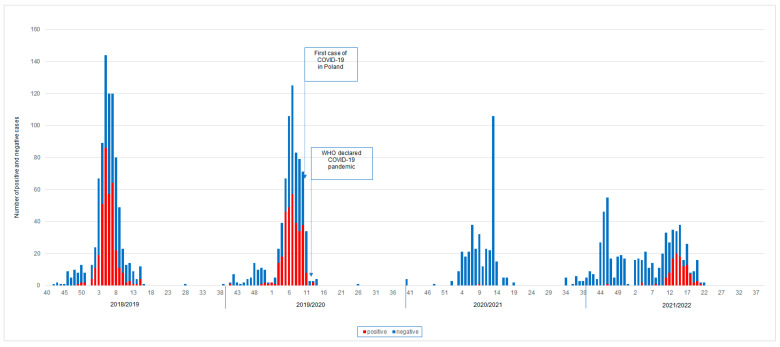
Weekly analysis of the number of positive and negative samples in the last four epidemic seasons.

In the 2018/2019 and 2019/2020 epidemic seasons, the peak incidence was recorded in the sixth week of 2019 and the eighth week of 2020, respectively (Figure 1). In the analyzed seasons, a maximum of 144 and 125 samples were tested per week, respectively. In the 2020/2021 season, the situation changed. The number of tested samples decreased. The largest number of tested samples was reported only in week 13 (*n* = 106), while in the remaining weeks the number did not exceed 40 samples per week. There was only one positive sample at week 9. During the epidemic season of 2021/2022, the number of positive samples remained at a similar level between week 13 and week 15 of the year in question. The peak incidence was recorded at week 14.

### 3.2. Percentage of Positive Samples

**Figure 2:**
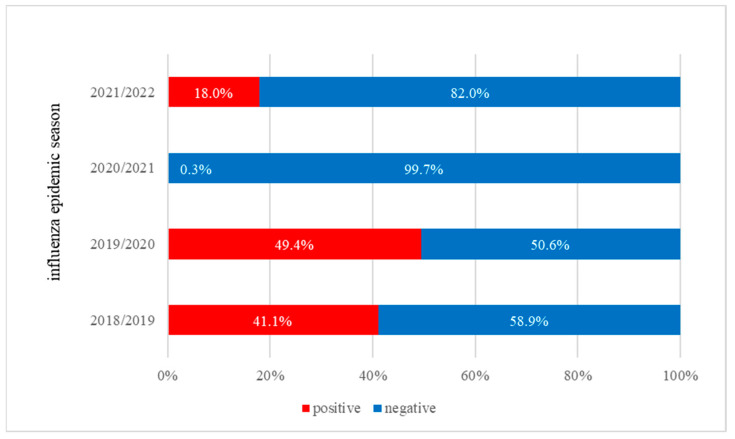
Percentage of positive and negative samples in the last four epidemic seasons.

In the 2018/2019 and 2019/2020 seasons, it was observed that the percentage of positive samples remained at a similar level and fluctuated between 41–49.4% (Figure 2). However, during the 2020/2021 season, there was a radical change in the tendency. Positive samples constituted only 0.3% of all tested specimens. In the 2021/2022 season, negative trials accounted for over 80% of all tested trials. The percentage of positive samples was much higher than in the 2020/2021 influenza season, but has not reached a level comparable to that before the pandemic.

### 3.3. Predomination of Influenza Types and Subtypes

**Figure 3:**
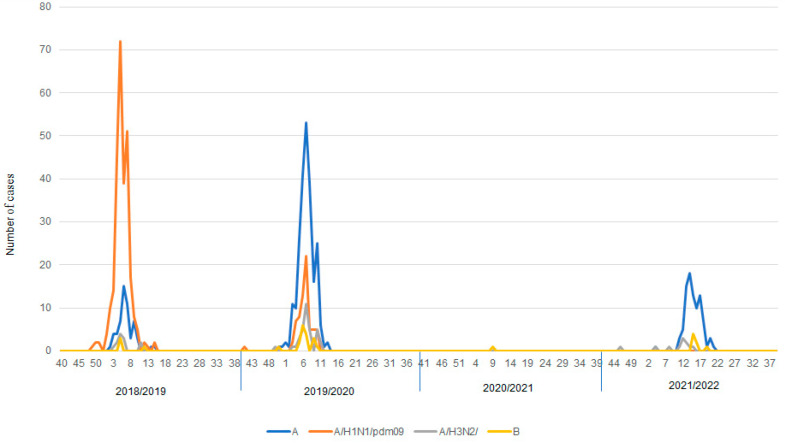
Predomination of influenza types and subtypes in the last four epidemic seasons.

The data was also analyzed in terms of the predominant influenza type in each epidemic season. Influenza A dominated in the epidemic seasons of 2018/2019, 2019/2020 and 2021/2022 (Figure 3). When analyzing the predominant subtypes in an epidemic season, it can be observed that in the case of seasons 2018/2019 and 2019/2020, the dominant subtype was influenza A/H1N1/pdm09. On the other hand, in the 2021/2022 season, influenza A/H3N2/prevailed. There was no case of influenza A/H1N1/pdm09 during whole epidemic season. Only one positive sample was recorded in the 2020/2021 epidemic season. It was a case of the influenza B infection. Influenza B remained at a low level in all analyzed seasons.

### 3.4. Averaged Data from Epidemic Seasons before and after Pandemics

**Figure 4:**
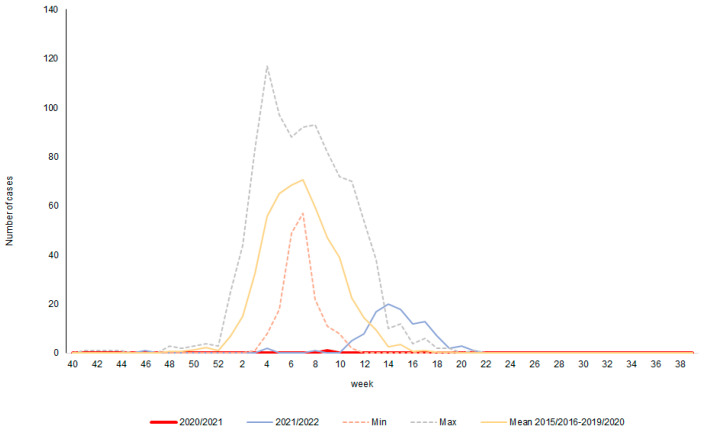
Comparison of averaged data from epidemic seasons before and after pandemics.

The average number of positive samples for the 2015/2016–2019/2020 epidemic seasons was also compared with the situation after the pandemic in the 2020/2021 and 2021/2022 epidemic seasons. The obtained results indicate an evident change of the trend. Only one positive test was observed in the 2020/2021 season. In the epidemic season of 2021/2022, this trend was slightly reversed, with positive samples recorded more often (between weeks 10 and 21 of the year) (Figure 4). However, the number of positives in relation to the mean was significantly lower. It was also lower than the lowest values recorded in the previous epidemic seasons. The activity of influenza was much delayed compared to the seasons before the pandemic.

### 3.5. Age Groups

**Figure 5:**
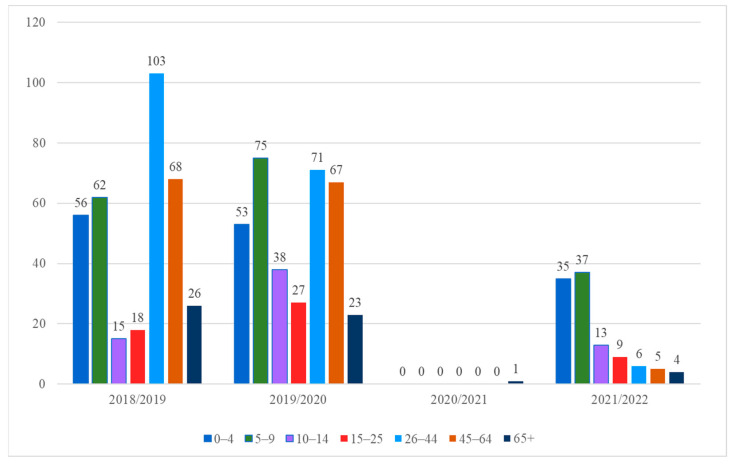
The number of positive tests divided into seven age groups in four epidemic seasons.

The positive tests (divided into seven age groups) were analysed. In the 2018/2019 season, the highest number of positive tests was recorded in the 26–44 age group (*n* = 103) (Figure 5). In the 2019/2020 season, a similar number of positive samples were recorded in the 5–9 (*n* = 75), 26–44 (*n* = 71) and 45–64 (*n* = 67) age groups. In the 2020/2021 epidemic season, only one positive sample was recorded. The patient was over 65 years of age. In the 2021/2022 season, a clear change in trends became visible. The highest number of positive tests was recorded in the 0–4 age group (*n* = 35) and the 5–9 age group (*n* = 37). In the case of the rest of the positive tests, their number decreased with the age of the patients.

### 3.6. Voivodships

**Figure 6:**
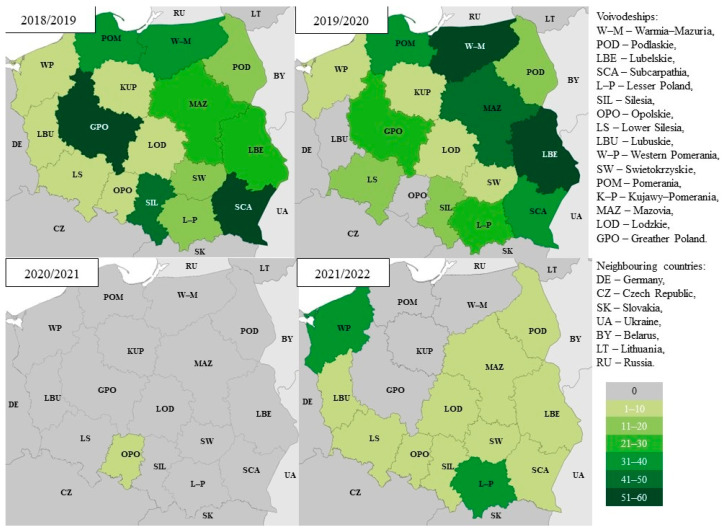
Comparison of positive samples in the last four epidemic seasons divided into voivodships in Poland.

In the 2018/2019 epidemic season, the largest number of positive samples was recorded in Greater Poland (*n* = 65) and Subcarpathia (*n* = 57), which accounted for 19% and 16% of all positive tests, respectively. In the western part of the country, in border voivodeships, only single positive tests were recorded (Western Pomeranian *n* = 1, Lubuskie *n* = 2, Lower Silesia *n* = 10, Opolskie *n* = 6) (Figure 6). In the 2019/2020 epidemic season, as in the 2018/2019 season, there was a difference in the number of test attempts compared to the west and east of the country. The highest number of positive samples was recorded in Warmia-Mazuria (*n* = 59). These tests accounted for 17% of all positive tests in the country. A large number of trials was recorded in Lubelskie (*n* = 52). They accounted for 15% of all positive tests in the country. In the west of the country, there were two voivodeships where no positive test was recorded throughout the epidemic season: Lubuskie and Opolskie. A difference in the number of positive tests can be seen in the 2020/2021 epidemic season. Throughout the season, there was only one positive test, which was reported in the Opolskie Voivodeship. In the 2021/2022 epidemic season, single positive tests were recorded in 10 out of the 16 voivodeships, and their number did not exceed 10. In four voivodships, no positive tests were recorded (Pomerania, Warmia-Mazuria, Kujawy-Pomerania, and Greater Poland). In the Western Pomerania and Lower Silesia Voivodeships, 31 positive tests were recorded in each season, and these were the highest values recorded for all voivodeships in the country.

## 4. Discussion

In 2020, the SARS-CoV-2 coronavirus pandemic began. A high number of lockdowns and the fact that people worked remotely resulted in a decrease in the incidence of infectious diseases, including influenza. The course of the 2020/2021 epidemic season was definitely different from the trend in the previous seasons. Low influenza activity was confirmed until September 2021 [16]. An increase in the incidence of respiratory diseases was not observed until the early spring of 2021 [17].

Thanks to the limitation on interpersonal contacts, the influenza epidemic season was shortened by about 6 weeks, which resulted in fewer hospitalizations and deaths due to influenza [18]. In the United States, influenza cases declined and remained historically low [17]. The decline in cases is not unique to influenza. It was estimated that in the US, RSV cases had dropped by 20% since the start of the COVID-19 pandemic [19]. This was most likely due to the minimization of interpersonal contacts, including the transition to remote work and distance learning, or restrictions on the use of public transport. The obligatory use of protective masks and the use of disinfectants also had a significant impact on reducing the number of cases. While in Asia the wearing of masks has been promoted since the SARS epidemic in 2003 [20], they had not been used by Western countries on a daily basis. Those habits changed as a result of the COVID-19 pandemic. As a consequence of the fact that influenza is spread by droplets, the use of face masks in everyday life has helped to reduce the spread its spread [21]. In Poland, the requirement to wear masks was lifted on 28 March 2022 [22].

Another reason for the lower number of reported influenza cases since the beginning of the COVID-19 pandemic may the result of changes in healthcare systems [23]. Each country was forced to adapt to the new reality. Healthcare systems were overloaded, which disrupted both the routine influenza testing of patients and the overall work of the surveillance network.

An interesting change that could be observed in the epidemic season of 2021/2022 in Poland was the delay in the activity of the influenza virus. This corresponds to the trends which can also be noticed in other countries in both the northern and southern hemispheres. In the USA, the peak of the epidemic season in 2021/2022 took place in spring, where the end of the epidemic season was observed earlier in the same period [23]. Variations in the circulation of the influenza virus were also noted in the countries of the southern hemisphere. Brazil reported unusual flu virus activity between October and November 2021 [24].

It is also interesting that changes in the circulation of influenza B were observed. Until the outbreak of the pandemic, two lineages of influenza B were circulating: B/Yamagata and B/Victoria. However, based on data from the Global Initiative on Sharing All Influenza Data (GISAID) database, it was determined that, since March 2020, no cases of influenza type B/Yamagata lineage have been reported worldwide [25]. There is a possibility that the sequences were not good enough to be included in the database. However, after the decrease in the number of cases of influenza B/Yamagata during a few epidemic seasons, we can conclude that it was not only the pandemic that could have had an impact. This may have been influenced by the introduction of quadrivalent vaccines: strains A/H1N1/pdm09, A/H3N2/, B/Victoria lineage and B/Yamagata lineage) (2012) [26]. Previously, only trivalent vaccines were used (strains A/H1N1/pdm09, A/H3N2/and, depending on the season, the B/Victoria lineage or the B/Yamagata lineage).

The comparison of Poland against the background of the Sentinel surveillance system in Europe is also interesting. In the 2018/2019 epidemic season in Europe, the co-dominance of A/H1N1/pdm09 (39%) and A/H3N2/ (33%) could be observed, while the influenza B virus accounted for only 0.9% of all positive tests [27]. In Poland, the A/H1N1/pdm09 virus clearly dominated. The underestimation of the number of A/H3N2/cases may be due to the fact that some positive tests for influenza type A were not subtyped, and VSESs often do not distinguish the A/H3N2/subtype, as this subtype is not included in most commercial tests. In the 2019/2020 season, influenza A accounted for 66% of positive tests, while influenza B accounted for 34% of them [28]. A slight increase in the number of influenza B cases could also be seen in Poland. In Europe, the co-dominance of subtypes A/H1N1/pdm (51%) and A/H3N2/ (49%) was again noted [28]. In Poland we can observe the dominance of influenza type A without specific subtypes. This situation suggests that a large proportion of the samples were subtype A/H3N2/. For the 2020/2021 season, only 10 positive tests were reported across Europe [29]. In Poland, only one positive sample was recorded. The trend reversed in the 2021/2022 season, when more than 4000 positive tests were reported across Europe, which accounted for 13% of the tested samples [30]. In Poland, positive tests accounted for 18.5% of the tested samples. In Europe, subtype A/H3N2/was clearly dominant (91.5% of all cases of influenza A) [30]. In Poland, a clear dominance of non-subtyped samples was noticeable, which, as in the 2019/2020 season, suggested the dominance of the A/H3N2/subtype.
